# Effect of Collagen and GelMA on Preservation of the Costal Chondrocytes’ Phenotype in a Scaffold *in vivo*

**DOI:** 10.17691/stm2023.15.2.01

**Published:** 2023-03-29

**Authors:** E.V. Isaeva, A.A. Kisel, E.E. Beketov, G.A. Demyashkin, N.D. Yakovleva, T.S. Lagoda, N.V. Arguchinskaya, D.S. Baranovsky, S.A. Ivanov, P.V. Shegay, A.D. Kaprin

**Affiliations:** Senior Researcher, Laboratory of Tissue Engineering; A. Tsyb Medical Radiological Research Centre — Branch of the National Medical Research Radiological Centre of the Ministry of Health of the Russian Federation, 10 Zhukova St., Obninsk, 249036, Russia;; Researcher, Laboratory of Tissue Engineering; A. Tsyb Medical Radiological Research Centre — Branch of the National Medical Research Radiological Centre of the Ministry of Health of the Russian Federation, 10 Zhukova St., Obninsk, 249036, Russia;; Researcher, Laboratory of Medical and Environmental Dosimetry and Radiation Safety; A. Tsyb Medical Radiological Research Centre — Branch of the National Medical Research Radiological Centre of the Ministry of Health of the Russian Federation, 10 Zhukova St., Obninsk, 249036, Russia; Associate Professor, Engineering Physics Institute of Biomedicine; Obninsk Institute for Nuclear Power Engineering — Branch of the National Research Nuclear University MEPhI, 1 Studgorodok, Obninsk, 249034, Russia;; Head of the Department of Pathomorphology; A. Tsyb Medical Radiological Research Centre — Branch of the National Medical Research Radiological Centre of the Ministry of Health of the Russian Federation, 10 Zhukova St., Obninsk, 249036, Russia; Head of Department of Histology and Immunohistochemistry, Institute of Translational Medicine and Biotechnology; I.M. Sechenov First Moscow State Medical University (Sechenov University), 8/2 Malaya Trubetskaya St., Moscow, 119991, Russia;; Lecturer; Medical Technical School, 75 A Lenina St., Obninsk, 249037, Russia;; Research Laboratory Assistant, Laboratory of Tissue Engineering; A. Tsyb Medical Radiological Research Centre — Branch of the National Medical Research Radiological Centre of the Ministry of Health of the Russian Federation, 10 Zhukova St., Obninsk, 249036, Russia;; Junior Researcher, Laboratory of Tissue Engineering; A. Tsyb Medical Radiological Research Centre — Branch of the National Medical Research Radiological Centre of the Ministry of Health of the Russian Federation, 10 Zhukova St., Obninsk, 249036, Russia;; Head of Laboratory of Tissue Engineering; A. Tsyb Medical Radiological Research Centre — Branch of the National Medical Research Radiological Centre of the Ministry of Health of the Russian Federation, 10 Zhukova St., Obninsk, 249036, Russia; Researcher, Research and Educational Resource Center for Cellular Technologies; Peoples’ Friendship University of Russia, 6 Miklukho-Maklaya St., Moscow, 117198, Russia;; Corresponding Member of the Russian Academy of Sciences, Director; A. Tsyb Medical Radiological Research Centre — Branch of the National Medical Research Radiological Centre of the Ministry of Health of the Russian Federation, 10 Zhukova St., Obninsk, 249036, Russia; Professor, Department of Oncology and X-ray Radiology named after V.P. Kharchenko, Medical Institute; Peoples’ Friendship University of Russia, 6 Miklukho-Maklaya St., Moscow, 117198, Russia;; Head of the Center for Innovative Radiological and Regenerative Technologies; National Medical Research Radiological Center of the Ministry of Health of the Russian Federation, 4 Koroleva St., Obninsk, 249036, Russia; Professor, Academician of the Russian Academy of Sciences, General Director; National Medical Research Radiological Center of the Ministry of Health of the Russian Federation, 4 Koroleva St., Obninsk, 249036, Russia Head of the Department of Urology and Operative Nephrology with a Course of Oncourology, Medical Institute; Peoples’ Friendship University of Russia, 6 Miklukho-Maklaya St., Moscow, 117198, Russia;

**Keywords:** scaffold, chondrocyte phenotype, micromolding, type I atelocollagen, GelMA, cartilage tissue

## Abstract

**The aim of the study** was to compare type I collagen-based and methacryloyl gelatin-based (GelMA) hydrogels by their ability to form hyaline cartilage in animals after subcutaneous implantation of scaffolds.

**Materials and Methods:**

Chondrocytes were isolated from the costal cartilage of newborn rats using 0.15% collagenase solution in DMEM. The cells was characterized by glycosaminoglycan staining with alcian blue. Chondrocyte scaffolds were obtained from 4% type I porcine atelocollagen and 10% GelMA by micromolding and then implanted subcutaneously into the withers of two groups of Wistar rats. Histological and immunohistochemical studies were performed on days 12 and 26 after implantation. Tissue samples were stained with hematoxylin and eosin, alcian blue; type I and type II collagens were identified by the corresponding antibodies.

**Results:**

The implanted scaffolds induced a moderate inflammatory response in both groups when implanted in animals. By day 26 after implantation, both collagen and GelMA had almost completely resorbed. Cartilage tissue formation was observed in both animal groups. The newly formed tissue was stained intensively with alcian blue, and the cells were positive for both types of collagen. Cartilage tissue was formed among muscle fibers.

**Conclusion:**

The ability of collagen type I and GelMA hydrogels to form hyaline cartilage in animals after subcutaneous implantation of scaffolds was studied. Both collagen and GelMA contributed to formation of hyaline-like cartilage tissue type in animals, but the chondrocyte phenotype is characterized as mixed. Additional detailed studies of possible mechanisms of chondrogenesis under the influence of each of the hydrogels are needed.

## Introduction

Cartilage is a dense connective tissue with limited self-recovery capabilities [1]. Highly organized extracellular matrix (ECM) of a cartilage consists mainly of proteoglycans and collagens [2]. Regeneration of various types of cartilage is complex and requires development of a microenvironment that facilitates formation of cartilage tissue of hyaline, elastic, or fibrous type [3]. Regenerative medicine has a means to restore local cartilage defects in the form of implantation of autologous chondrocytes [4]. The process requires cells obtained from healthy areas of cartilage tissue and propagated *in vitro* [4-6]. The method’s disadvantages include the limited number of chondrocytes and their tending to dedifferentiation during 2D cultivation, as well as possible damage to the donor area [7-9].

The technology of tissue engineering using scaffolds based on natural hydrogels provides new possibilities for repairing cartilage defects. Hydrogels are a network of cross-linked hydrophilic polymers. In water, hydrogel swells, and its mass increases in a large degree. Physical and biochemical properties of hydrogels largely depend on hydrogel composition, polymerization methods, and crosslinking density. Hydrogels may serve as a universal platform with the desired combination of properties to be used in regenerative medicine and other related areas [10]. For instance, biomedical hydrogels are designed to reproduce characteristics of native ECM and provide three-dimensional support to dividing cells during the tissue formation process [11]. The advantages of natural hydrogels include better biocompatibility and minimal inflammatory response in the body [12-14]. Their disadvantage is poor mechanical properties, resulting in the need to add other components (materials) that increase rigidity, or apply various methods of polymerization of the base material.

Another important matter relates to the extent to which hydrogels can regulate cell phenotype in tissue engineering structures. Ideally, hydrogel should facilitate chondrogenesis and regeneration of complex area-based organization of native cartilage, whereas the newly formed matrix should feature the original hyaline cartilage rich in type II collagen and aggrecans [15]. From this point of view, it is promising to study the capability of various hydrogels to support chondrogenesis *in vitro* and after implantation of chondrocyte-containing scaffolds into animals. Type I collagen [16-21] and methacryloyl gelatin (GelMA) [9, 22–25] are used in scaffolds for cartilage tissue. However, the results are often quite contradictory.

Collagen is the main component of ECM. It is a fibrillar protein that is part of various forms of connective tissue, such as bones, cartilage, tendons, and skin [26– 29]. In nature, type I collagen exists in the form of a triple helix (α1)2β2 [30, 31]. Being the most common ECM protein, collagen contains cell adhesion sites based on the arginine–glycine–aspartic acid (RGD) tripeptide and is characterized by low immunogenicity [32]. Collagen and its derivatives are most often used as bio-inks in bioprinting and basis for scaffolds in general, especially in work with cells [33, 34].

Gelatin is a protein substance containing denatured and partially hydrolyzed native collagen, mainly type I collagen [35]. Thermal denaturation reduces gelatin antigenicity compared to collagen [36]. Here, bioactive collagen sequences (for example, RGD) for cell attachment and matrix metalloproteinase-sensitive sites responsible for cell-mediated degradation are preserved [37]. Gelatin modified with methacryloyl (methacrylamide or methacrylate) side groups (GelMA) increasingly spreads in tissue engineering, as together with good biocompatibility it has better in quality and widely tuned mechanical properties, especially compared with other available gel-forming biomaterials [38, 39]. Introduction of photo-crosslinkable methacryloyl substituent groups provides convenient and rapid gel formation under UV-irradiation in the presence of photoinitiators [40]. Application of this material for three-dimensional cell cultivation not only imitates natural extracellular environment, but also provides creation of well-defined tissue structures [9, 23].

**The aim of the study** was to compare hydrogels based on type I collagen and GelMA as to their ability to support formation of hyaline cartilage in animals after subcutaneous implantation of scaffolds.

## Materials and Methods

### Cell culture

The primary chondrocyte culture was obtained according to the previously described protocol [20]. Nine 5-day-old rat pups were taken to isolate cells from costal cartilages. Soft tissues were removed by incubation in 0.25% trypsin (PanEco, Russia) and 0.2% type I collagenase (Gibco, USA) solutions under moderate stirring on a magnetic stirrer (Biosan, Latvia) at +37°С. Costal cartilages in a 0.15% collagenase solution were left overnight at +37°C in a CO_2_ incubator (Sanyo, Japan). Uncleaved tissue fragments were removed by means of filtration (100 nm, nylon; SPL Lifesciences, South Korea). To inactivate enzyme, fetal bovine serum (Biosera, Brazil) was added and washed by centrifugation (Elmi, Latvia). After the last centrifugation, cell pellet was resuspended in a DMEM nutrient medium (glucose content of 4.5 g/L); then, 200 μl of the suspension was sampled, stained with 0.4% trypan blue solution in phosphate-buffered saline (pH 7.4; ECO-Service, Russia) and then the number of living cells was counted.

The cells were placed in bottles with a surface area of 225 cm2 (Corning, USA) in a DMEM nutrient medium (glucose content of 4.5 g/L) added with 10% fetal bovine serum (Biosera, Brazil), penicillin and streptomycin (50 IU/ml and μg/ml, respectively), and glutamine (649 μg/ml). The cells were cultivated for 7 days until a monolayer (~90%) was formed, after which it was removed from plastic and used for mixing with hydrogels. The affiliation of cells with cartilage tissue was determined by formation of glycosaminoglycans (GAGs) in accordance with the previously described method [19].

### Collagen-based hydrogel

Hydrogel was prepared using a sterile solution of type I porcine atelocollagen (80 mg/ml; Imtek, Russia) in line with the previously described method [41]. On the day of experiment, chondrocytes were taken off the plastic with a trypsin:EDTA solution (1:1); after staining with trypan blue, the number of living cells was counted, centrifuged (400 g, 5 min), and resuspended in 0.25 ml of DMEM medium (glucose content of 4.5 g/L) without serum. The cell suspension was added with 0.25 ml of collagen buffer (Tris-HCl; 0.3 M; pH 8.0) and cooled at 4°C for 10 min. This solution was mixed with 0.5 ml of collagen solution, thus receiving a hydrogel with the collagen concentration of 4%. The final concentration of cells amounted to ~20**·**10^6^ m/L^–1^. The hydrogel was kept at 4°C until the use.

### GelMA-based hydrogel

GelMA (Bloom 300; Sigma-Aldrich, USA; 900496) was used for GelMA-based hydrogel. First, 6 mg of Irgacure 2959 photoinitiator (Sigma-Aldrich; 410896) was weighed and placed in 600 μl of sterile distilled water. Then, it was dissolved at 70°C for 10–15 min in Termite solid-state thermostat (DNA-Technology, Russia), after which it was cooled in the dark at room temperature. The cooled solution was added to 80 mg of pre-weighed GelMA and left overnight at 4°C to swell. On the next day, the solution was kept in a thermostat for 3–4 h at 40°C, subject to periodical shaking, until GelMA was completely dissolved. Chondrocytes were taken off from plastic, and after staining with trypan blue, the number of living cells was counted, centrifuged, and resuspended in 200 μl of DMEM medium without serum. This volume of solution was mixed with 600 μl of GelMA, thus obtaining a hydrogel with a gelatin concentration of 10%. The final concentration of cells amounted to ~30**·**10^6^ m/L^–1^. This hydrogel was kept at 37°C until use.

### FDM printing

**Figure 1.**
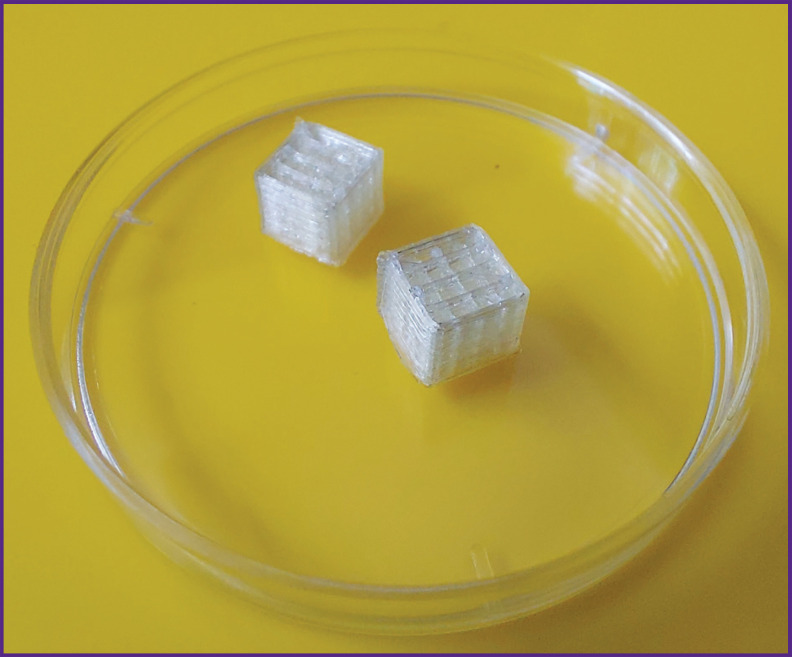
PVA mold appearance

One day before the experiment, cube-shaped frames with the dimensions of 5×5×5 mm and an internal lattice were printed from PVA (polyvinyl acetate, filament diameter of 1.75 mm) using the FDM (fused deposition modelling) method (Figure 1). The cell size was 1×1×1 mm. Printing was conducted on a Rokit INVIVO 3D bioprinter (Rokit, South Korea), software version 1.68. The model was sliced with the NewCreatorK software, version 1.57.63. Printing was made to a flat unheated printing table, the extruder operating temperature was 200°C. The thickness of one layer was 0.2 mm, with a nozzle diameter of 0.2 mm. Before printing, the skirt was applied to provide the proper first layer. Also, the inner parts of the printer were sterilized before printing using a built-in 254-nm UV-lamp. The finished frames were UV-sterilized with the same wavelength for 2 h.

### Preparation of tissue engineering structures (scaffolds)

Scaffolds were made by micromolding. On the day of the experiment, 12 PVA frames were placed in sterile Petri dishes (Corning, USA) of 35 mm in diameter, 3 pieces in each dish. Then, 6 frames were filled with chilled collagen-based hydrogel, added with warm nutrient medium, and placed in a CO_2_ incubator (Sanyo, Japan) (37°C, 5% CO_2_) for polymerization. The remaining 6 frames were placed on an ice pack, filled with warm GelMA-based hydrogel, kept for 30 min, then filled with cold nutrient medium, and irradiated with ultraviolet light (365 nm) in the internal space of the printer for 10 min at a temperature of the printing table of 0…+4°C. After irradiation, the frames were kept for another 30 min in cold, then placed in a CO_2_ incubator (Sanyo, Japan).

**Figure 2.**
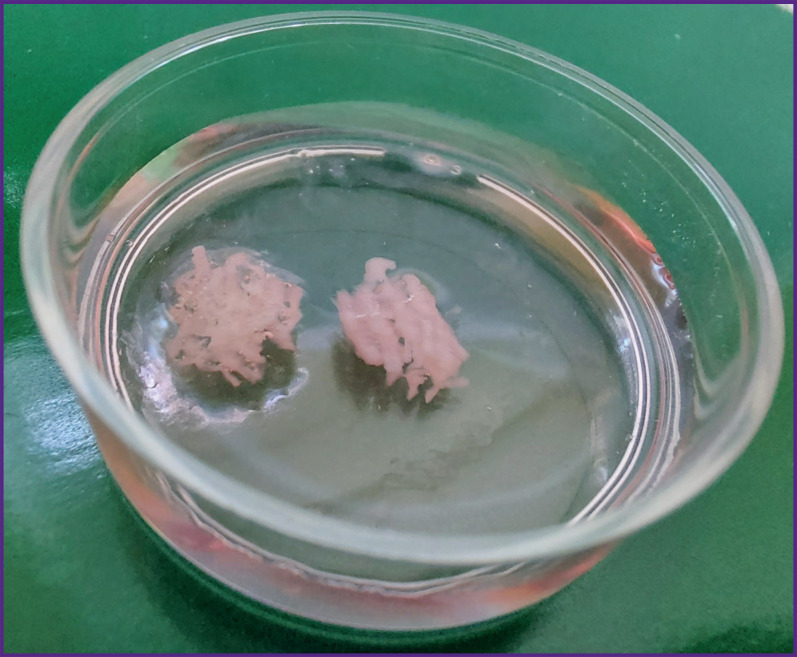
Appearance of the scaffolds after the PVA mold dissolution

A few hours later, when the PVA frames were subjected to dissolution, the obtained scaffolds were washed with phosphate-buffered saline to remove PVA remains, placed in new sterile Petri dishes, added with DMEM medium (glucose content — 4.5 g/L) and 10% fetal bovine serum, and left in the incubator until the next day. After polymerization of hydrogels and PVA dissolution, the finished structures had a lattice form (Figure 2).

### Scaffold implantation to animals

After approximately 18 h of incubation, the scaffolds were implanted subcutaneously in the withers of eight 2-month-old male Wistar rats weighing ~200 g. The animals were divided into two groups of 4 rats. The rats in group 1 were implanted with collagen-based scaffolds, the animals in group 2 — with GelMA-based scaffolds. All procedures were performed using inhalation ether anesthesia (Chimmed, Russia). Animals were shaved off at the implantation site, the surgical field was treated with 70% ethanol solution (RFK, Russia), an incision was made with scissors, and a pocket where the implant was placed was formed under the skin with a scalpel. The wound was sutured with the edges of the pocket tightened, the site of implantation was marked with a colored thread of suture material (Monocryl Poliglecaprone 25; Ethicon, USA). The suture was treated with a 3% hydrogen peroxide solution. A better fixation was achieved by application of medical glue (Vertex, Russia) on top of the suture. The area around the surgical field was additionally anesthetized with 0.5% Novocain solution (Dalkhimpharm, Russia). During the experiment, the rats were isolated from each other in one-place cages with free access to water and food. The surgical field was controlled daily. Two animals from each group were euthanized with an interval of ~2 weeks — on day 12 and day 26 after implantation — and the material was taken for histological and immunohistochemical analyses. All work with laboratory animals was conducted in accordance with the European Convention for the Protection of Vertebrate Animals used for Experimental and Other Scientific Purposes and approved by the local bioethics commission.

### Histological and immunohistochemical analyses

Scaffolds with fragments of surrounding tissues were fixed for 24 h in Bouin’s acidic fluid: 1.3% trinitrophenol (Sigma-Aldrich, USA) and 40% formalin (BioVitrum, Russia). After washing in 70% ethanol, a standard histological testing of the samples was performed, followed by their placement in the Histomix paraffin medium (BioVitrum). Paraffin sections of 5 μm were sliced on a microtome (Leica RM 2235; Leica Microsystems, Germany) and placed on silanized glass slides (S3003; Dako, Denmark). For histological analyses, deparaffinized sections were stained with hematoxylin, eosin and alcian blue (8GX; Sigma-Aldrich). After alcohol dehydration and clarification in ortho-xylene, the samples were put in Canadian balsam (Merck, Germany).

Immunohistochemical studies were conducted using monoclonal rabbit antibodies to type II collagen (SAB4500366, 1:50; Sigma-Aldrich) and polyclonal rabbit antibodies to type I collagen (FNab01836, 1:100; Fine Test, China). To immunovisualize rabbit antibodies, secondary goat IgG rabbit antibodies were conjugated with horseradish peroxidase (ab205718, 1:1000; Abcam, USA). Immunohistochemical solutions were prepared in phosphate-buffered saline. According to the protocol for immunohistochemical studies, deparaffinized sections immersed in citrate buffer (pH 6.0) were boiled for 5 min before primary antibodies to type I and II collagen were applied. Endogenic peroxidase was blocked in 3% hydrogen peroxide solution. 2% normal serum of animal donors of secondary antibodies, 1% bovine serum albumin and 0.1% Triton X-100 were added to the blocking buffer. The samples were incubated overnight in the solution of primary antibodies in a humid chamber at +4°C. After washing the preparations in phosphate buffer, secondary goat rabbit antibodies were applied to the sections for 1 h at room temperature. Diaminobenzidine (Liquid DAB+, K3468; Dako North America, Inc., USA) was used to identify substrate peroxidase. After alcohol dehydration and xylene clearing, the preparations were put into Canadian balsam. Histological sections were examined under an Axio Imager A1 microscope (Carl Zeiss, Germany) with microphotography on the Canon Power Shot A640 digital camera (Canon, Japan).

## Results

### Isolation of cells from costal cartilages

**Figure 3.**
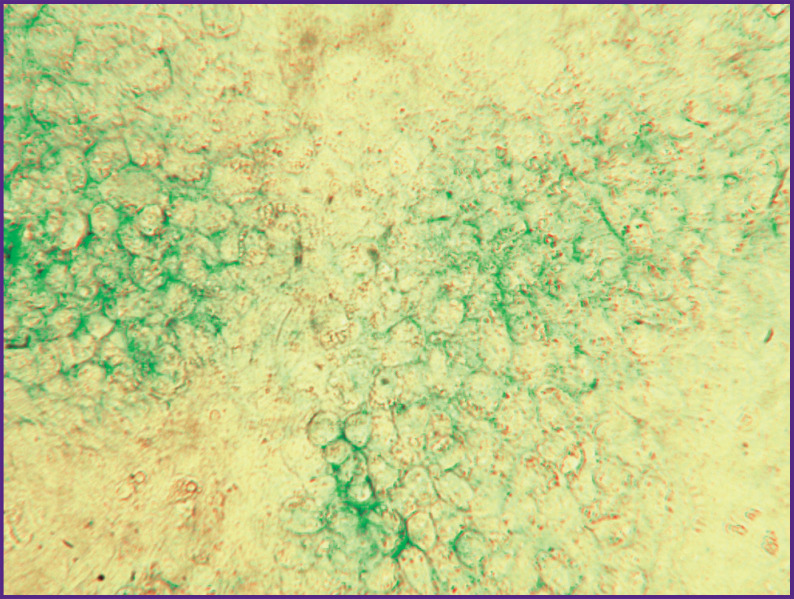
Chondrocytes of the 0^th^ passage after 7 days of cultivation in a Petri dish Alcian blue staining, glycosaminoglycans; vol. ×25

During the primary isolation from the costal cartilages of newborn rats, the number of cells was ~16.5 million, in which the content of living cells was 93.9%. Zero passage cells had a cubic shape and granular cytoplasm typical of chondrocytes; they formed a dense monolayer on day 7 of cultivation. Alcian blue staining revealed specific metabolites of chondrocytes, GAGs, in the intercellular space (Figure 3), which was the basis to identify isolated cells as cartilaginous. The viability of zero passage cells taken off from the plastic on the day of the experiment before mixing with hydrogels was ~95%.

### Study in animals

#### Group 1 (type I atelocollagen), day 12 after implantation

**Figure 4.**
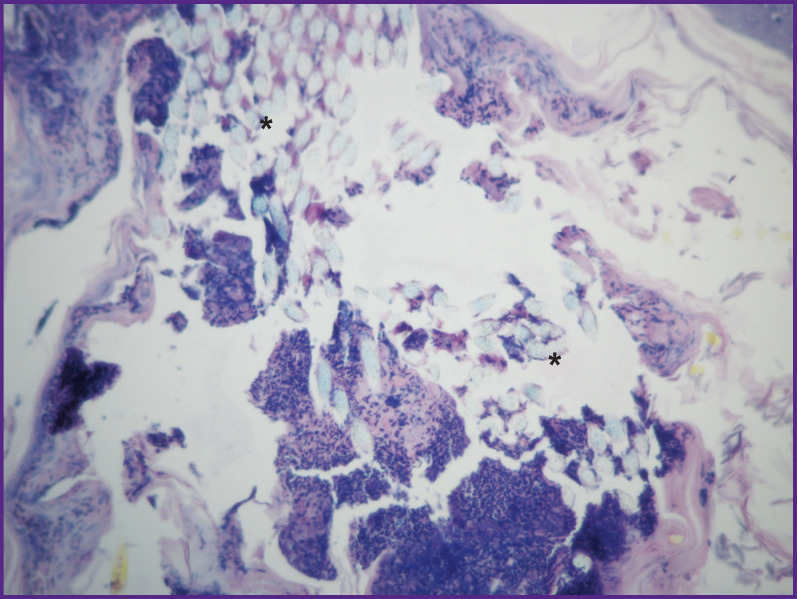
Group 1 (atelocollagen-based scaffolds); day 12 after implantation Necrotic tissues in the implant area; staining with hematoxylin and eosin; vol. ×20. Asterisks mark the remains of the suture thread that point to the implantation site

**Figure 5.**
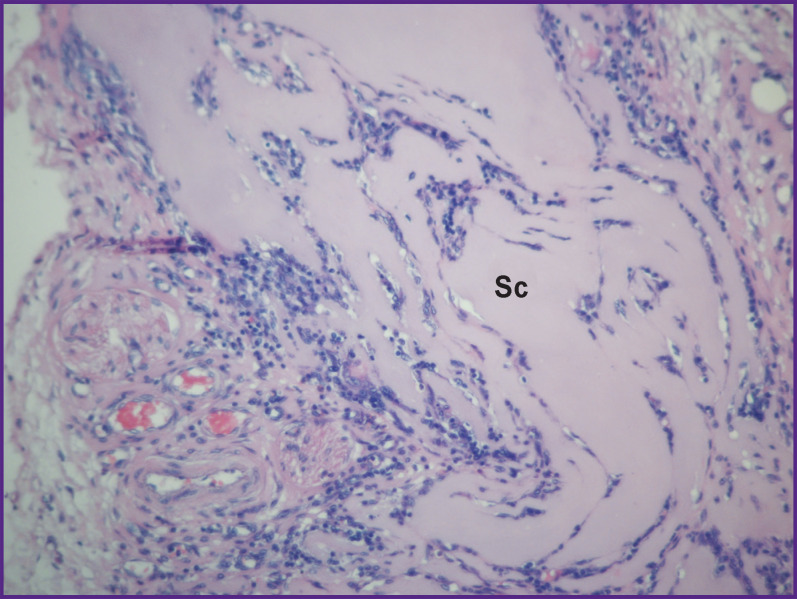
Group 1 (atelocollagen-based scaffolds); day 12 after implantation Scaffold is surrounded by a connective tissue capsule — Sc; staining with hematoxylin and eosin; vol. ×20

**Figure 6.**
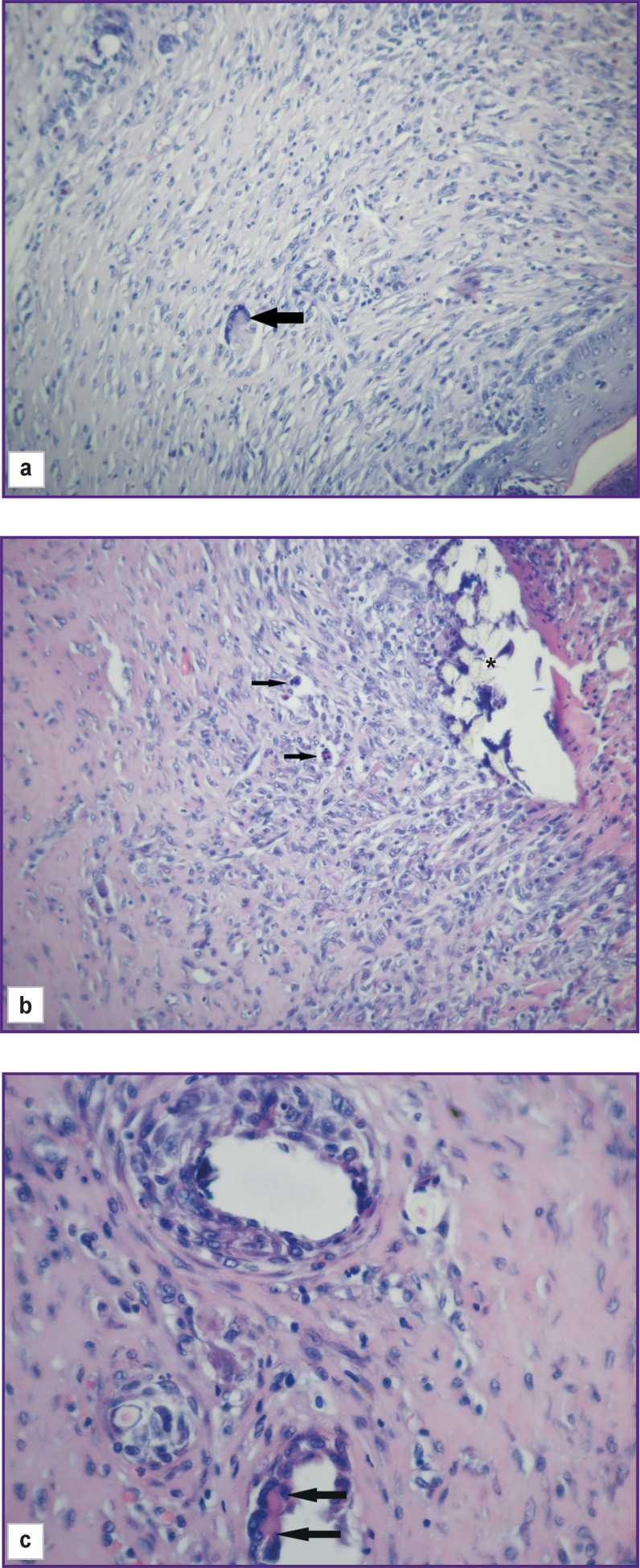
Group 1 (atelocollagen-based scaffolds); day 12 after implantation Connective tissue capsule around the scaffold: (a) and (b) connective tissue with multinuclear cells of resorbed foreign bodies (*marked with arrows on figure a–c*); an asterisk indicates the remains of the suture thread at the implantation site. Staining with hematoxylin and eosin; vol. ×20; (c) is a part of figure (b); vol. ×40

**Figure 7.**
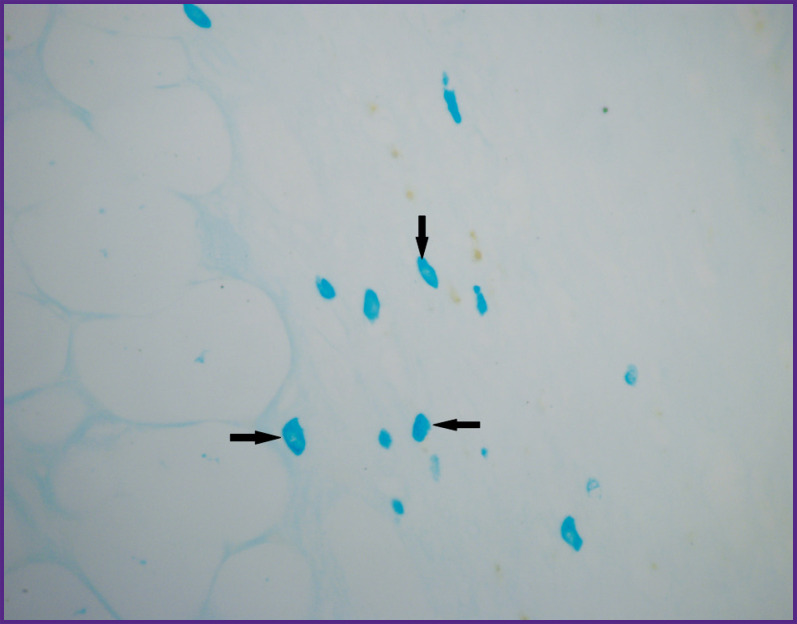
Group 1 (atelocollagen-based scaffolds); day 12 after implantation Mast cells in the connective tissue capsule around the implant (*shown by arrows*). staining with alcian blue; vol. ×40

At this point, the site of scaffold implantation is identified by presence of remains of the suture thread and necrotic tissues on histological preparations of the skin with subcutaneous adipose tissue and muscles taken from animals (Figure 4). The implant is represented by bundles of collagen fibers separated by connective tissue cells growing into it (Figure 5). By this time, a moderately overgrown connective tissue capsule was formed, in which multinuclear macrophages and foreign body resorption cells are seen (Figure 6). There are large cells along the blood vessels in the subcutaneous tissue (together with elements of fibrous connective tissue); the cytoplasm of these large cells shows an intense reaction with alcian blue, which makes it possible to classify these cellular elements as granule cells, considering their location and intense staining of the cytoplasm in the acidic environment (Figure 7). Collagen staining was not performed at this point. There were no signs of cartilage tissue formation.

#### Group 1 (type I atelocollagen), day 26 after implantation

**Figure 8.**
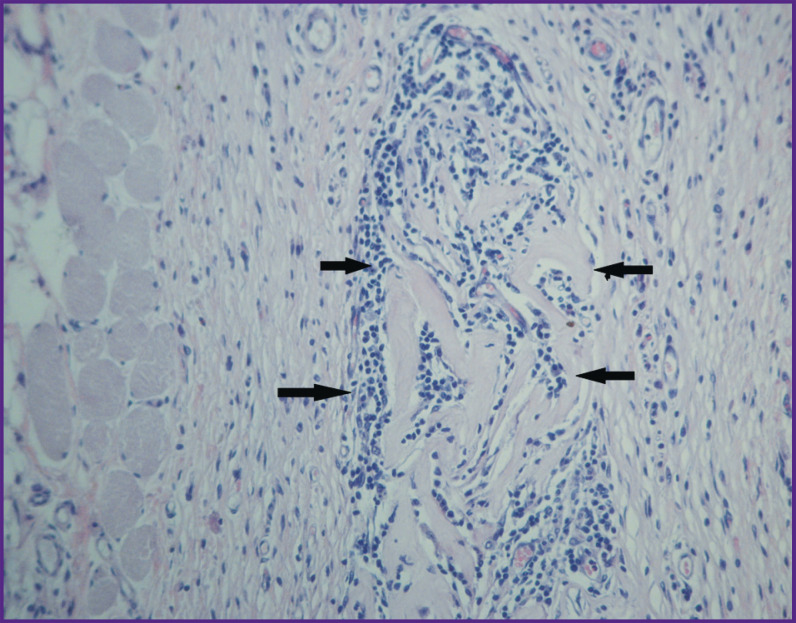
Group 1 (atelocollagen-based scaffolds); day 26 after implantation Fragments of the implant lattice (*shown with arrows*). Staining with hematoxylin and eosin; vol. ×20

**Figure 9.**
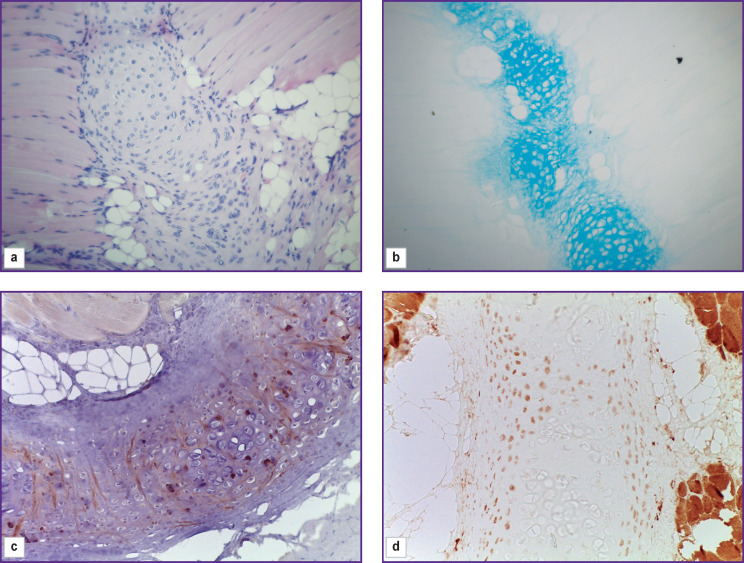
Group 1 (atelocollagen-based scaffolds); day 26 after implantation An island of cartilage tissue in the vicinity of the scaffold: (a) staining with hematoxylin and eosin; (b) staining with alcian blue; (c) immunohistochemical reaction to type II collagen; (d) immunohistochemical reaction for type I collagen; vol. ×20

**Figure 10.**
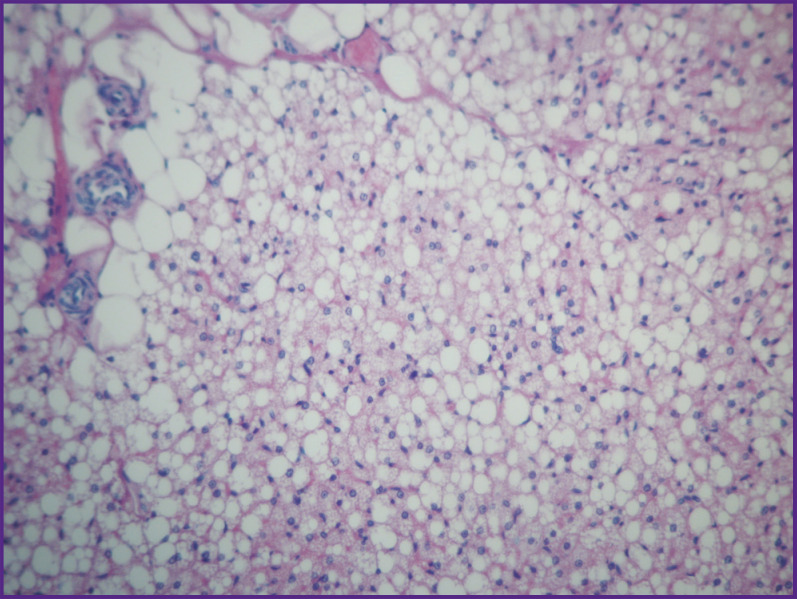
Group 1 (atelocollagen-based scaffolds); day 26 after implantation Brown fat (multidroplet lipocytes) in the rat subcutaneous tissue. Staining with hematoxylin and eosin; vol. ×20

At this point, scaffold remains are identified in the implantation area, they are divided into small fragments by the round cell infiltrate (Figure 8). Multinuclear macrophages are detected in the connective tissue capsule. One of the animals demonstrated foci of proliferation of cartilage tissue among the striated muscles in the implantation area (Figure 9 (a)), which was stained with alcian blue (Figure 9 (b)) and chondrocytes from which gave a positive immunohistochemical reaction for type II collagen (Figure 9 (c)). In cells located closer to the perichondrium, type I collagen was also found (Figure 9 (d)). Pronounced growth in the implantation area of brown fat is to be paid attention to (Figure 10).

#### Group 2 (GelMA), day 12 after implantation

**Figure 11.**
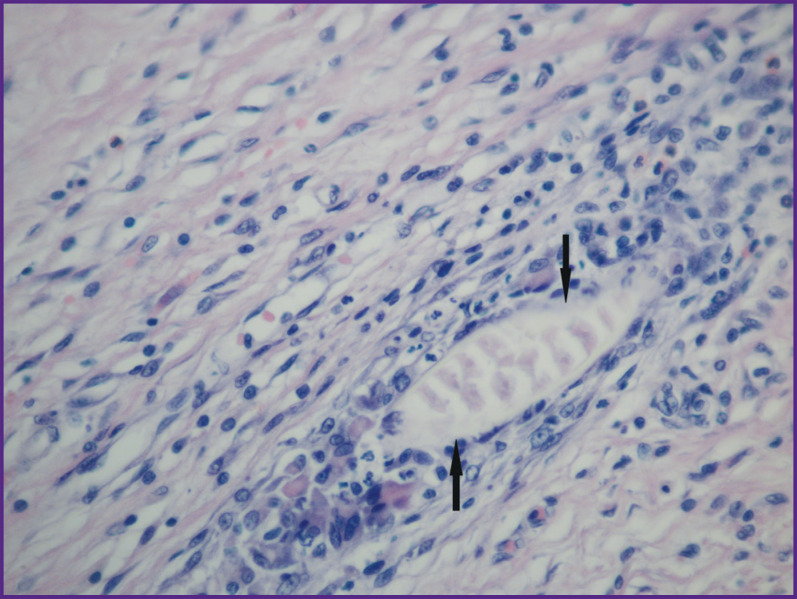
Group 2 (GelMA-based scaffolds); day 12 after implantation Fragments of the implant lattice in the rat subcutaneous tissue (*shown with arrows*). Staining with hematoxylin and eosin; vol. ×40

**Figure 12.**
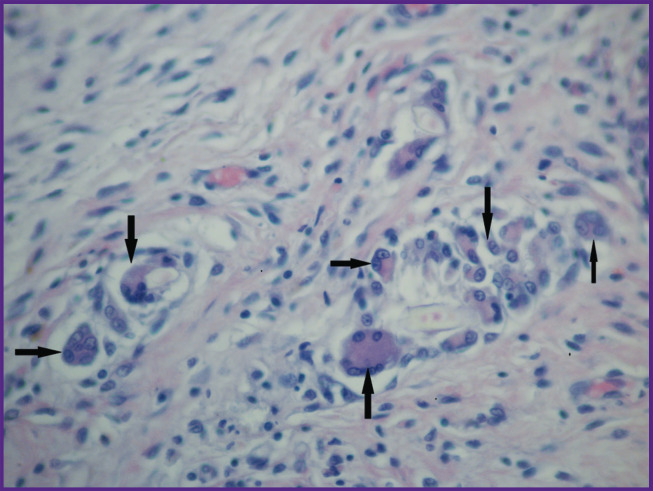
Group 2 (GelMA-based scaffolds); day 12 after implantation Multinuclear cells of foreign bodies resorption in the connective tissue capsule (*shown with arrows*). Staining with hematoxylin and eosin; vol. ×40

**Figure 13.**
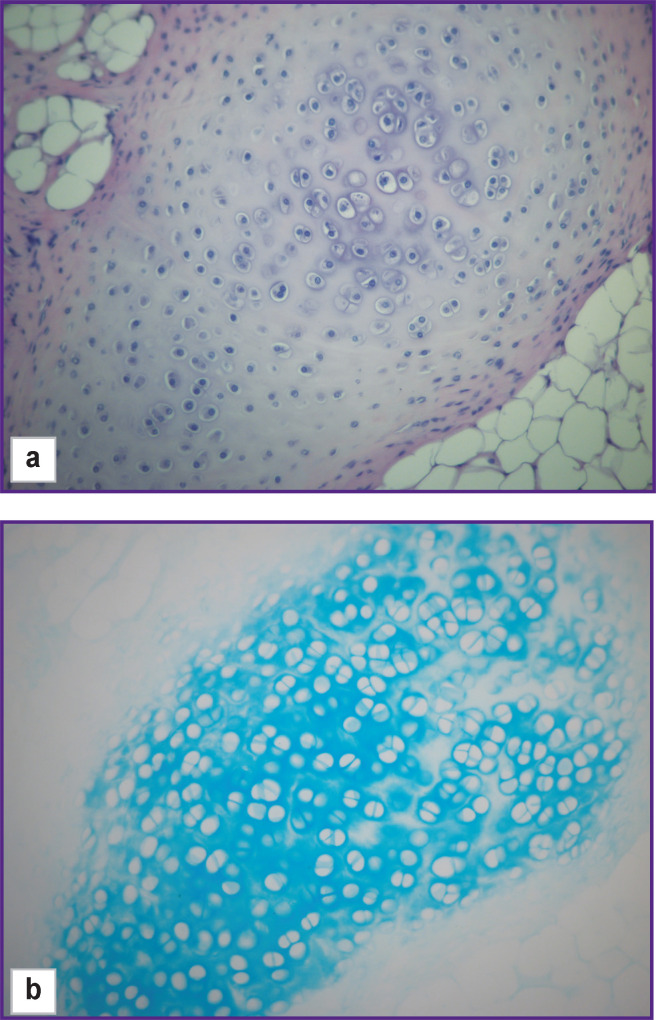
Group 2 (GelMA-based scaffolds); day 12 after implantation Cartilage tissue at the implant site: (a) staining with hematoxylin and eosin; (b) staining with alcian blue; vol. ×20

At this point, fragments of randomly located fibers were noted in the implant location area of euthanized animals (Figure 11). There were few multinuclear cells of resorption of foreign bodies in the neighboring connective tissue (Figure 12). Along the blood vessels in the subcutaneous connective tissue, large cells are found, the cytoplasm of which is intensely stained with alcian blue (similar to animals in group 1). One of the animals demonstrated an island of cartilage tissue among the muscle fibers in the implantation area (Figure 13 (a)), intensely stained with alcian blue (Figure 13 (b)). Collagen staining was not performed at this point.

#### Group 2 (GelMA), day 26 after implantation

**Figure 14.**
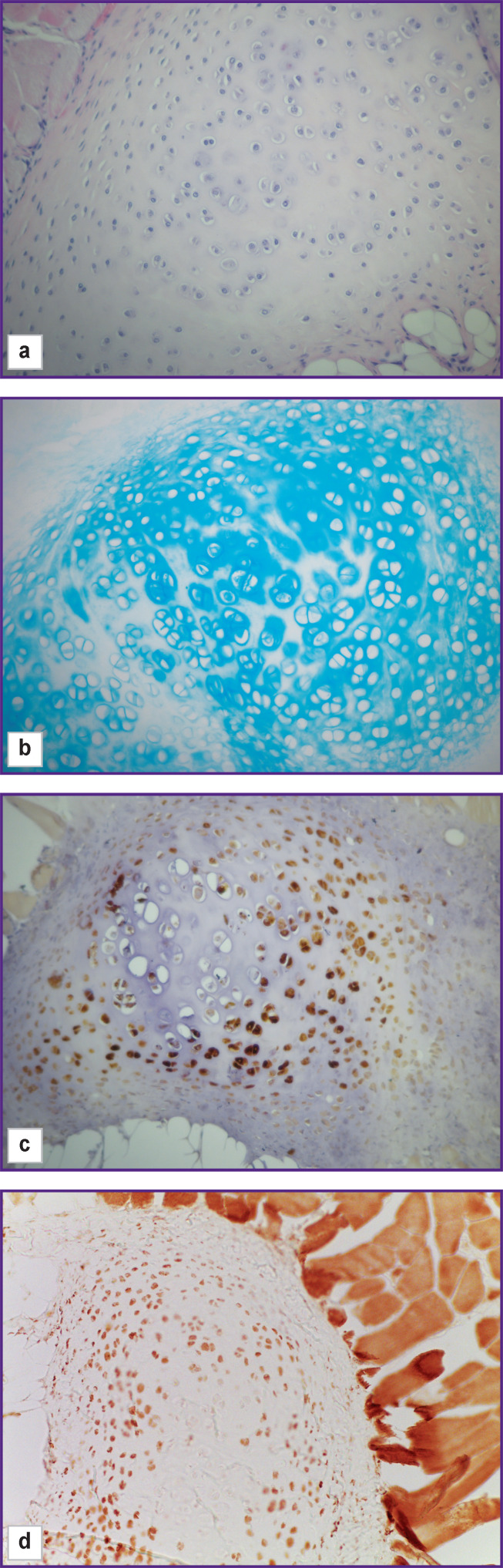
Group 2 (GelMA-based scaffolds); day 26 after implantation Cartilage tissue at the implant site: (a) staining with hematoxylin and eosin; (b) staining with alcian blue; (c) immunohistochemical reaction to type II collagen; (d) immunohistochemical reaction for type I collagen; vol. ×20

At this point, an island of cartilaginous tissue with a poorly developed perichondrium was found among the striated muscles of euthanized animal (Figure 14 (a)). The cartilage consists of sparsely located chondroblasts, sole as a rule, which do not form isogenic groups. The cytoplasm of cartilage cells and small areas of the territorial matrix show an intense reaction when stained with alcian blue (Figure 14 (b)). Immunohistochemical staining revealed a high content of type II collagen in cytoplasm of chondrocytes located closer to the perichondrium (Figure 14 (c)); type I collagen was also found in the same cells (Figure 14 (d)).

## Discussion

This study covered the ability of hydrogels based on natural polymers, type I collagen and GelMA, to preserve the costal chondrocyte phenotype and stimulate chondrogenesis *in vivo*. It is known that three-dimensional scaffolds not only serve as a substrate for cell attachment, ECM synthesis, and accumulation of bioactive molecules, but also can direct cell differentiation by influencing interactions between cells, as well as between cells and ECM [42]. Such scaffolds can support repeated differentiation of dedifferentiated chondrocytes both *in vitro* [43] and *in vivo* [38].

To preserve the shape of the obtained scaffolds and make mechanical properties of the structures similar to those of native cartilage, we took hydrogels with a high concentration of collagen (4%) and GelMA (10%). A hydrogel with such a concentration of collagen was already used in our previous studies related to tissue engineering [20, 21, 44] and demonstrated its suitability for such purposes. Increasing the polymer concentration is the simplest approach to immediately increasing the density and rigidity of hydrogel. However, diffusion of metabolites can be difficult in dense hydrogels without additional channels [38, 44]; therefore, tissue-engineered structures were made in the form of a lattice to reduce the diffusion distance. Micromolding is a fast and cost-effective approach to production of hydrogel-based scaffolds [40].

PVA is a water-soluble, highly swelling, non-toxic, biocompatible [45] and biodegradable polymer with good adhesive properties [46], which allows to use it in experiments with cells. The method used to get scaffolds provided satisfactory results: after the PVA dissolution, the scaffold lattice was preserved, whereas the access of nutrients to the cells inside hydrogels could be implemented from several sides.

When implanted in animals, the scaffolds caused a moderate inflammatory response in both groups, which is evidenced by the macrophages and granule cells presence in the surrounding connective tissue capsule. It is known that exposure to pro-inflammatory cytokines can stimulate chondrocyte migration [47, 48]. By day 26 after implantation, both collagen and GelMA were almost completely resorbed. Cartilage tissue, as in the previous studies [20, 21], was formed among muscle fibers, which can be explained by a good blood supply of muscle tissue.

Type II collagen and GAG are considered specific markers of hyaline cartilage, which are detected by alcian blue staining in an acidic environment [15]. Their qualitative detection in histological and immunohistochemical analyses allows to conclude that one animal in group 1 with implanted atelocollagen-based scaffolds demonstrated formation of hyaline-like cartilage tissue on day 26 after implantation. In animals in group 2, which were implanted with GelMA-based scaffolds, hyaline-type cartilage tissue was formed both on day 12 and day 26 after implantation. We are far from believing that this result is only due to the scaffold material, as in the previous study [20], cartilage tissue was formed in atelocollagen-based scaffolds with rat costal chondrocytes in all experimental animals during the observation period from day 5 to day 40. Quantitative assessment of the GAG content was not conducted because, according to available data, infiltration of host cells into the implanted scaffold can distort the results of *in vivo* studies [38]. The detection of type I collagen in the cytoplasm of chondrocytes may be primarily related to immaturity of the newly formed cartilage tissue on day 26 after implantation of the scaffolds. Sophia Fox et al. [2] noted that, in addition to type II collagen, which accounts for 90–95% of ECM, hyaline cartilage contains types I, IV, V, VI, IX, and XI collagens. It is possible that the expression of type I collagen by chondrocytes would have been insignificant later in the study.

Many studies show that hydrogels based on pure or modified type I collagen supported chondrogenesis both *in vitro* [43, 49] and *in vivo* [50]. At the same time, other results were also published. For instance, Jin and Kim [51] stated that rat joint chondrocytes cultured in a chondrogenic medium in a gel based on type I collagen (concentration of 3.87 mg/L were dedifferentiated in contrast to the same chondrocytes in gels based on alginate and alginate-collagen mixture, which kept a specific phenotype. Schlegel et al. [52] also showed that chondrocytes can take a fibroblast-like (flattened) shape in type I collagen hydrogels.

Gelatin, being a product of collagen denaturation, biochemically has little differences from the above. However, methacryloyl gelatin contains methacrylamide or methacrylate functional groups subjected to photo-crosslinking under UV-irradiation in the presence of a photoinitiator. Tissue engineering structures based on methacryloyl gelatin demonstrate that both the source of gelatin and the type of photoinitiator can affect repeated differentiation of chondrocytes [53], and irradiation of cells decreases their viability [54]. The results obtained for this hydrogel are also contradictory. Daly et al. [3] stated that hydrogels based on GelMA and type I collagen in a chondrogenic medium *in vitro* promoted synthesis of matrix components corresponding to fibrous cartilage, whereas agarose and alginate stimulated development of hyaline-like cartilage tissue by bone marrow mesenchymal stem cells. Schuurman et al. [23] demonstrated that GelMA-based hydrogels maintained viability and differentiated state of encapsulated articular equine chondrocytes during cultivation *in vitro* for 4 weeks. Boere et al. [38] investigated the ability of human joint chondrocytes to synthesize specific products of cartilage tissue in GelMA scaffolds reinforced with a mixture of poly(hydroxymethylglycolide-co-ε-caprolactone)/ poly(ε-caprolactone) polymers. It was shown that encapsulated chondrocytes demonstrated significant production of cartilage matrix in these structures both *in vitro* and *in vivo*. Levett et al. [15] reported that GelMA-based hydrogels of low elasticity (1.5 kPa) supported cell proliferation and GAG-enriched matrix accumulation. Here, chondrocytes were of a mixed phenotype with high expression of type I collagen. Based on this finding, the authors conclude that it is necessary to apply several indicators of cartilage formation simultaneously, as the widely used indicators, such as GAG production and alcian blue staining, do not necessarily correlate with *in vitro* chondrogenesis.

In this study, the histological analysis showed that after the implantation of scaffolds both collagen and GelMA contributed to formation of hyaline-like cartilage tissue in the animal bodies. At that, chondrocytes of the newly formed tissue expressed both type II collagen and type I collagen. It should be noted that the *in vivo* environment is very complex and, thus, can differently affect the production of ECM components and scaffold degradation, which complicates direct comparison of the results with those of *in vitro* studies. Based on the data received, one can conclude that chondrocytes of the newly formed cartilage tissue on day 26 after scaffolds implantation in animals were of a mixed phenotype.

## Conclusion

The available published data on the ability of hydrogels based on type I collagen and GelMA to support hyaline cartilage formation are often conflicting. In this study, both collagen and GelMA contributed to formation of hyaline-like cartilage tissue in animals on day 26 after scaffold implantation, but chondrocytes were of a mixed phenotype. Considering that each material has certain advantages and disadvantages, profound studies of potential chondrogenesis mechanisms under the influence of each hydrogel type are needed.
